# STING agonist inflames the pancreatic cancer immune microenvironment and reduces tumor burden in mouse models

**DOI:** 10.1186/s40425-019-0573-5

**Published:** 2019-04-29

**Authors:** Weiqing Jing, Donna McAllister, Emily P. Vonderhaar, Katie Palen, Matthew J. Riese, Jill Gershan, Bryon D. Johnson, Michael B. Dwinell

**Affiliations:** 1 Department of Medicine, Milwaukee, USA; 2 Department of Microbiology & Immunology, Medical College of Wisconsin, 8701 Watertown Plank Road, Milwaukee, WI 53226 USA; 3 MCW Center for Immunology, Milwaukee, USA; 4 Department of Pediatrics, Medical College of Wisconsin, Milwaukee, USA; 5 Cell Therapy Laboratories, Medical College of Wisconsin, 8701 Watertown Plank Rd, Milwaukee, WI USA

**Keywords:** Cytotoxic T cells, Tumor infiltrating lymphocytes, CXCL10, Cancer proliferation, Tumor selectivity, STING agonist, Immune activation

## Abstract

Pancreatic cancer is characterized by an immune suppressive stromal reaction that creates a barrier to therapy. A murine transgenic pancreatic cancer cell line that recapitulates human disease was used to test whether a STimulator of Interferon Genes (STING) agonist could reignite immunologically inert pancreatic tumors. STING agonist treatment potently changed the tumor architecture, altered the immune profile, and increased the survival of tumor-bearing mice. Notably, STING agonist increased numbers and activity of cytotoxic T cells within tumors and decreased levels of suppressive regulatory T cells. Further, STING agonist treatment upregulated costimulatory molecule expression on cross-presenting dendritic cells and reprogrammed immune-suppressive macrophages into immune-activating subtypes. STING agonist promoted the coordinated and differential cytokine production by dendritic cells, macrophages, and pancreatic cancer cells. Cumulatively, these data demonstrate that pancreatic cancer progression is potently inhibited by STING agonist, which reignited immunologically cold pancreatic tumors to promote trafficking and activation of tumor-killing T cells.

**Electronic supplementary material:**

The online version of this article (10.1186/s40425-019-0573-5) contains supplementary material, which is available to authorized users.

## Background

Pancreatic ductal adenocarcinoma (PDA) is the fourth leading cause of cancer deaths with an overall 5-year-survival rate of only 8%, the lowest of any cancer [1, 2]. Surgical resection is considered necessary for the long-term survival of patients with this treatment refractory disease, yet over 80% of patients don’t meet surgical candidacy criteria upon diagnosis [3]. Of the patients who do undergo surgery, more than 70% will die from recurrent disease owing in part to the lack of success from standard-of-care chemotherapy regimens, including gemcitabine or FOLFIRINOX [3]. Finding effective therapeutics remains challenging because the unique tumor microenvironment of PDA tumors, composed of dense extracellular matrix, fibroblast cells and immunosuppressive leukocytes, drives therapeutic resistance and creates an immunologically tolerant space [4–7].

Transformation of normal cells into malignant neoplasms results in cancer cells escaping from immune detection, immune evasion, or suppressing immune responses against the mutated cells [4]. The cancer-immunity cycle is a multistage process needed to overcome these tumor evasion and suppression strategies [8]. Key to an effective anti-tumor immune response is the priming and activation of T cells by antigen-presenting cells, followed by directed trafficking and infiltration of T cells into the tumor mass so that they recognize and kill cancer epithelial cells [8]. Two broadly-defined categories describe immune microenvironments within tumors; the first is T cell-inflamed, or “hot” tumors, while the second is non-T cell-inflamed, or “cold” tumors [9, 10]. In hot tumors, immune evasion mechanisms likely act at the effector T cell phase since tumor antigen-reactive T cells have already homed to the tumors but lose their ability to kill tumor cells [11]. Tumor cells often upregulate programmed death ligand-1 (PD-L1), whose inhibitory checkpoint receptor, PD-1, is expressed on tumor antigen-specific T cells within the tumor microenvironment (TME). Engagement of PD-LI with PD-1 on T cells promotes immune tolerance within the tumor [12]. In cold tumors, where tumor antigen-specific effector T cells are deficient, immune evasion strategies likely arise earlier and interfere with both immune priming and trafficking stages [13]. As such, immune checkpoint blockade therapy has been a successful treatment modality in highly immunogenic “hot” tumors but has shown little efficacy in non-immunogenic “cold” tumors such as pancreatic cancer [14–16]. Indeed, despite the presence of tumor-reactive lymphocytes in peripheral immune tissues and serum, this notoriously immune-suppressive carcinoma is largely devoid of tumor-reactive immune cells [17–21]. A pre-existing T cell-driven immune response to PDA is likely necessary for effector phase immune-modulating therapies to be effective [21, 22]. Strategies to activate the innate immune system have recently shown promise in re-engaging non-immunogenic tumors to drive anti-cancer immunity [23–25].

Innate immune activation as a booster for generating anti-cancer adaptive immunity has recently been employed in pancreatic cancer. An attenuated strain of *Listeria monocytogenes* expressing the pancreatic tumor-associated antigen mesothelin, in combination with GVAX, a vaccine produced from allogeneic whole pancreatic cancer cells expressing granulocyte-macrophage colony-stimulating factor (GM-CSF), has shown a survival benefit in patients [26–28]. This treatment regimen shifted the tumors toward a more immunogenic state as evidenced by increased T cell infiltration and presence of intra-tumoral tertiary lymphoid aggregates [29]. Innate immune cells utilize pattern recognition receptors to activate inflammatory signaling cascades upon binding to pathogen- or damage-associated molecular patterns. Cyclic GMP-AMP synthase (cGAS) is a cytoplasmic pattern recognition receptor that produces cyclic GMP-AMP (cGAMP) following recognition and binding of prokaryotic or eukaryotic double-stranded DNA. Stimulator of Interferon Genes (STING), a four-transmembrane spanning endoplasmic reticulum protein binds cGAMP and upregulates transcriptional gene programs within the cell, which ultimately results in type I interferon (IFN) production [30, 31]. Type I IFNs (IFNα and IFNβ) are required for the generation of antitumor CD8^+^ T cells. A type 1 interferon transcriptional signature has been associated with “hot” T cell-inflamed tumors [32, 33]. Activation of STING by systemic or intra-tumoral administration of STING agonists stimulates reversion of immune-suppression and tumor regression in multiple preclinical cancer models [34–39]. Therefore, activation of the STING innate immune sensing pathway shows promise to activate immune suppressed tumors by reverting tumor devoid of T cell infiltrates into tumors containing T cells activated against tumor antigens.

One of the most challenging aspects of tumor biology is overcoming immune suppression derived from systemic factors or cellular and soluble factors within TME. A dampening of T cell activation against tumor antigens as well as inhibition of T cell migration into the tumor is regulated by a myriad of suppressive factors. In this study, transgenic mouse models of pancreatic cancer were used to test the hypothesis that STING agonists could functionally activate anti-tumor immune reactivity. For these studies we used 5,6-dimethyl-9-oxo-9H-xanthene-4-acetic acid (DMXAA), a xanthenone analog also known as vadimezan or ASA404. DMXAA failed clinical trials and was subsequently shown to specifically activate murine STING signaling pathways [30, 31, 40]. We discovered that the murine STING agonist DMXAA increased the survival of pancreatic cancer-bearing mice. In the tumor, there was an increase in the production of inflammatory cytokines and chemokines that facilitate T cell migration, an upregulation of maturation markers on dendritic cells (DC), and an increase in the quantity and functional capacity of tumor infiltrating cytotoxic T cells. These data show that activation of innate immunity through the administration of STING agonist therapy can reverse tumor immune suppression in PDA.

## Methods

### Murine pancreas cancer cells

Two murine pancreatic cancer cell lines, FC1242 and FC1199, were kindly provided by the Tuveson laboratory (Cold Spring Harbor Laboratory, Cold Spring Harbor, NY). Hereafter referred to as KPC1242 and KPC1199 these murine pancreatic cancer cells were isolated from spontaneously arising tumors from KRas^LSL.G12D/+^-p53^R172H/+^-Pdx-Cre (KPC) transgenic mice on a homogenous C57BL6 background [41]. Murine pancreatic cancer cells were maintained in high-glucose DMEM and penicillin /streptomycin antibiotics (Life Technologies Inc., Carlsbad, CA, USA) with 10% (*v*/v) FBS (Omega Scientific, Tarzana, CA, USA) as previously described [41, 42] and hereafter referred to as complete growth medium.

### Orthotopic and subcutaneous syngeneic pancreas cancer models

For subcutaneous tumors, 1 × 10^6^ KPC1242 or KPC1199 cells were implanted in the right rear flank of C57BL/6J mice (Jackson Laboratory, Bar Harbor, Maine, USA). Male and female RAG1 knockout mice (B6.129S7-Rag1^tm1Mom^/J) were obtained from Jackson Lab and maintained in an in-house colony. Mice were sorted into vehicle or treatment groups and treated with DMXAA, 5,6-dimethyl-9-oxo-9H-xanthene-4-acetic acid, (Tocris, Minneapolis, MN, USA) at a final concentration of 450 μg in 50 μL by intra-tumoral (i.t.) injection as indicated. Tumor area (mm^2^) was measured daily by calipers and calculated using the formula length x width. Mice were euthanized and tumors removed to measure tumor volume (length x width x depth) and for further analysis. Some mice received an intra-peritoneal (i.p.) injection of 200 μg of anti-CD4 (clone GK1.5), anti-CD8 (clone 2.43), or anti-NK1.1 (clone PK136) neutralizing antibodies (BioXCell, West Lebanon, NH, USA) one day before an initial DMXAA treatment. For orthotopic syngeneic engraftment, C57BL/6J mice were anesthetized and 1 × 10^6^ KPC1242 cells injected directly into the pancreas as previously described [43, 44]. Seven days after orthotopic implantation mice were sorted into untreated control or experimental treatment groups. Mice were treated with DMXAA at 300 μg in 200 μL volume by i.p. injection. Mice were euthanized, and tumors removed for flow and multiplex immune profile analyses. Cytokines and chemokines within pancreatic tumors in vivo were detected using a Mouse Cytokine Array/Chemokine Array 31-Plex (Eve Technologies, Calgary, AB, Canada). All experiments using mice were done in accordance with a Medical College of Wisconsin Institutional Animal Care and Use Committee-approved protocol.

### In vitro activation of tumor-associated myeloid cells or pancreatic cancer epithelial cells

Bone marrow–derived macrophages or dendritic cells were generated from freshly isolated mouse bone marrow cells. Briefly, bone marrow was washed from femurs and tibias in a sterile manner, pooled, and mononuclear cells cultured in complete medium supplemented with 20 ng/mL M-CSF, to generate macrophages, or GM-CSF (PeproTech, Rocky Hill, NJ, USA), to differentiate dendritic cells (DCs) for 7 days, with medium refreshed on day 3 and day 5. On day 7 DMXAA was added to culture medium to a final concentration of 20 μg/mL for 18 h. Bone-marrow derived macrophage growth medium was supplemented 48 h with either 100 ng/mL lipopolysaccharide (LPS) to induce differentiation into M1-type cells or 10 ng/mL IL-4 to differentiate cells into M2-type macrophages. Supernatants were collected for multiplex (Eve Technologies) and cells stained for flow cytometry analyses.

A total 1 × 10^6^ KPC1242 cancer cells were cultured overnight in a 24-well plate, washed, and switched to serum-free medium. Eighteen hours later, the cells were treated with 10 μg/mL gemcitabine (Hospira, Inc., San Clemente, CA, USA) for 1 h. Cells were washed and fresh serum-free medium containing 100 μg/mL DMXAA was added for an additional 4 h. Cells were washed and incubated an additional 18 h at 37 °C with complete growth medium. Supernatants were collected for unbiased multiplex analysis (Eve Technologies) or sandwich ELISA to quantify secreted CXCL10, a T cell chemoattractant, or CCL20, a DC chemokine (R&D Systems, Minneapolis, MN, USA). Supernatants were collected and frozen at − 80 °C before protein detection and quantification in accordance with the manufacturer’s directions. Multiplex and ELISA measured cytokines and chemokines from a minimum of four biological replicates, with each sample assayed in triplicate as technical replicates.

### Tumor infiltrate analysis

Tumors were excised, weighed and measured. Approximately half of each dissected tumor was fixed 48 h in zinc formalin for histopathologic analysis. The remaining tumor sections were placed in PBS with 1% (*v*/v) FBS and mechanically minced. Minced tumors were placed in gentleMACS Dissociator with Tumor Dissociation Kit for mouse tissues (Miltenyi Biotec, San Diego, CA, USA) to isolate immune and tumor cell subsets in accordance with the manufacturer’s directions.

### Flow cytometry

The following monoclonal anti-mouse antibodies and flow cytometry reagents were obtained from eBioscience (San Diego, CA, USA): anti-CD4 (clone GK1.5), anti-CD8 (clone 53–6.7), anti-CD11c (clone n418), anti-CD11b (clone M1/70), anti-CD40 (clone 3/23), anti-CD62L (clone WEL-14), anti-CD64 (clone X54–5/7.1), anti-CD69 (clone H1.2F3), anti-CD80 (clone 16-10A1), anti-CD86 (clone GL1), anti-CD206 (clone C068C2), anti-Ly6G (clone 1A8), anti-Ly6C (clone HK1.4), anti-F4/80 (clone BM8), anti-CD103 (clone 2E7), anti-PD-1 (clone J43), anti-PD-L1 (clone M1H5), anti-Ki67 (clone 20Raj1), anti-granzyme B (clone GB11), anti-FoxP3 (clone FJK-16 s), and 7AAD staining solution. The following monoclonal antibodies and reagents were obtained from BD Bioscience (San Jose, CA, USA): anti-CD3 (clone 145-2c11), anti-CD45.2 (clone 104), anti-CD8 (clone 53–6.7), and anti-MHC Class II (clone 2G9), and 7AAD staining solution. Flow cytometric analysis was completed using a BD Biosciences LSRII or Fortessa X20 (Franklin Lakes, NJ, USA) flow cytometer, and resulting data analyzed FlowJo software (Ashland, OR, USA).

### Histology

Tumors were fixed 48 h in zinc formalin, processed, embedded in paraffin, and 4 μm sections placed onto glass slides by trained personnel in the Children’s Research Institute of the Children’s Hospital of Wisconsin Histology Core. Tissue sections were stained with hematoxylin and eosin as well as Masson’s trichrome dyes as described previously [45].

### Proliferation/apoptosis assay

To engineer KPC1242 cells stably expressing nuclear-localized red fluorescent protein (NR), parental cells were transduced with IncuCyte NucLight Red Lentivirus Reagent (Essen Bioscience, Ann Arbor, MI, USA) in accordance with the manufacturer’s directions. Pure clonal populations of KPC1242-NR cells were plated at 18,000 cells per well in a 96 well plate and placed into the IncuCyte S3 in vivo imaging instrument. Cells were treated 48 h later with 10 μg/mL gemcitabine for 1 h before being replaced with fresh medium containing 100 μg/mL DMXAA for an additional 4 h. Cells were washed and cultured an additional 52 h in complete growth medium containing 5 μM Caspase 3/7 Green Apoptosis Reagent in accordance with the manufacturer’s instructions (Essen Bioscience). Cellular morphology and fluorescence intensity quantified every 2 h using phase microscopy, and the red and green channels, respectively according to optimized protocols.

### IFN-γ enzyme-linked ImmunoSpot (ELISpot) assay

Tumor-reactive IFN-γ secreting CD8^+^ T cells were harvested from spleens, purified by immunomagnetic sorting, and immediately used in ELISPOT assays using the mouse IFN-γ ELISPOT Kit (BD Biosciences) as described previously [46].

### T cell killing assay

Pancreatic tumors were dissociated using the gentleMACS Dissociator as described above to isolate tumor-infiltrating lymphocytes (TILs). To expand TILs ex vivo, CD8 T cells isolated using CD90.2 magnetic beads (Miltenyi Biotec) were incubated at a ratio of 5:1 with irradiated K562 artificial antigen presenting cells engineered to express CD32 (FcRγII) and CD137L (41BB-L) and loaded with 16 μg/mL anti-CD3 (clone 145-2c11) and 2 μg/mL anti-CD28 (clone 37.51) antibody (BD Biosciences). TIL-K562 co-cultures were incubated in RPMI full growth medium supplemented with 10% (*v*/v) FBS, penicillin /streptomycin, 50 μM 2-mercaptoethanol, L-glutamine, and the proliferative cytokines IL-2, (5 U/mL), IL-7 (5 ng/mL), and IL-15 (5 ng/mL) purchased from (PeproTech). After 7–10 days in culture, expanded tumor reactive TILs will were incubated at ratios of 1:5, 1:10 and 1:20 with KPC1242 for 24 h and tumor cell killing measured using IncuCyte S3 (Essen BioScience).

### Immunoblotting

Pancreatic cancer cells were plated at 1 × 10^6^ cells per plate (60 mm) and grown overnight in complete growth medium. Cells were then serum-starved overnight and stimulated with LPS [1 μg/mL] or gemcitabine [10 μg/mL] for 1 h, washed, treated an additional 4 h with DMXAA [100 μg/mL], washed again, and incubated 24 h in full growth medium. After stimulation, cells were re-washed and lysed using RIPA buffer. Lysates were normalized for protein concentration, and 10 μg whole cell lysates were size separated using reducing SDS-PAGE, electro-transferred to PVDF membranes (Millipore, Burlington, MA USA) and probed using primary antibodies against phosphorylated or total TBK1, STAT6, IRF3, and GAPDH (Cell Signaling Technology, Danvers, MA, USA), followed by horseradish peroxidase-conjugated secondary antibody (GE Healthcare, Marlborough, MA, USA). Proteins were visualized by chemiluminescence with auto-exposure and quantified by densitometric analysis using the ChemiDoc Touch (BioRad, Hercules, CA, USA).

### Statistical analysis

All statistical analyses were performed using GraphPad Prism 7.0 (La Jolla, CA, USA). Unpaired statistical analyses were calculated using a Student’s t-test. Multiple comparisons between groups were analyzed using a one-way ANOVA. Multiple comparisons between groups were analyzed using a one-way ANOVA and a log-rank Mantel-Cox test to identify differences in survival between distinct experimental groups. Statistical significance was defined as *P* ≤ 0.05.

## Results

### Treatment with STING agonist and gemcitabine activates CD8^+^ T cells and induces pancreatic cancer regression

**Fig. 1:**
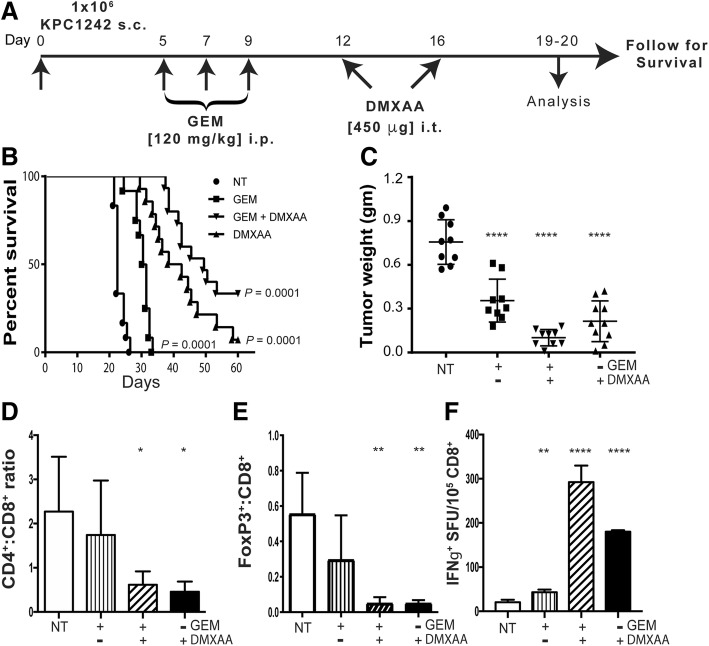
Increased survival and immune activation in mice treated with gemcitabine and STING agonist. **a** Experimental treatment strategy of subcutaneous (s.c.) pancreatic tumors in C57BL/6 mice. Control not treated (NT) and experimental mice were treated as indicated. **b** Kaplan-Meier survival curves are shown for the indicated control and experimental groups. Data are representative of 3 independent experiments (*n* = 14–15 mice per group). **c** Tumors were collected 19 or 20 days after implantation and tumor weight measured. **d**, **e** Tumors were processed into single cell suspensions and CD4:CD8 ratios and percent Foxp3^+^ cells within the CD8^+^ T cell compartment determined by flow cytometry. **f** Splenic CD8^+^ T cells were isolated by immunomagnetic sorting and tested in IFN-γ ELISPOT assays using KPC1242 tumor cells as stimulators. Values are mean IFN-γ spot forming unit (SFU) ± SD, *n* = 3 independent experiments. *, *P* ≤ 0.05; **, *P* ≤ 0.01; ***, *P* ≤ 0.001; ****, *P* ≤ 0.0001

Pancreatic cancers are uniquely characterized by a dense fibrotic matrix and the profound ability to avoid tumor-specific immune responses. Spontaneous tumors that arise in the KRas^G12D/+^, Trp53^R172H/+^, pdx-1-Cre (KPC) genetically engineered mouse model of PDA mirror human pancreatic cancer with elevated levels of immune suppressive macrophages and myeloid-derived suppressor cells, with concomitant decreased numbers of infiltrating T cells [17, 18, 47]. This unique immunological environment is maintained in tumors arising from either subcutaneous or orthotopic implantation of cell lines derived from KPC mice, including KPC1242 and KPC1199 cells [47, 48]. We therefore initiated studies using KPC syngrafts implanted subcutaneously in immunocompetent mice to model immune suppressed human pancreatic cancer. As single-agent immunotherapy has achieved limited clinical benefit to date in patients with PDA, we first assayed the potential for STING agonists to provide an additive effect when combined with standard-of-care cytotoxic chemotherapy. Gemcitabine, a nucleoside analog chemotherapy used for multiple cancers, exerts direct anti-tumor activity and may possess tumor immunotherapeutic effects [49, 50]. To test whether STING agonist and gemcitabine cooperated in controlling PDA growth, we treated KPC tumor-bearing mice with gemcitabine prior to administration of the murine STING agonist 5,6-Dimethyl-9-oxo-9H-xanthene-4-acetic acid (DMXAA) (Fig. 1a). Single agent gemcitabine treatment significantly delayed tumor progression (Fig. 1b) and resulted in a reduced tumor burden relative to non-treated tumor (Fig. 1c), with a ~ 1-week survival benefit in the KPC syngraft model. When intra-tumoral administration of DMXAA was initiated 12-days after tumor implantation, alone or in combination with gemcitabine, nearly all tumors regressed, resulting in significantly reduced tumor burden and smaller tumors upon analysis at days 19–20. The combination of gemcitabine and DMXAA treatment resulted in pronounced tumor regression and better survival, consistent with an additive effect of the dual treatment strategy (Fig. 1b, c). Notably, treatment with STING agonist alone significantly extended the survival time of tumor bearing mice. While survival was increased, tumor regression following two doses of STING agonist was transient, with recurrent tumors appearing within 10–14 days after the last injection (not shown).

We next asked if the regression and survival benefit observed in STING agonist treated mice reflected activation of intra-tumoral and/or systemic tumor-specific immune responses. Consistent with activation of adaptive immune responses, we observed a reversal in the CD4:CD8 T cell ratio, reflective of an increase in CD8^+^ lymphocytes within tumors treated with DMXAA, either alone or in combination with gemcitabine (Fig. 1d). Given that gemcitabine alone had a negligible effect on intra-tumoral CD4:CD8 levels, these data suggest the recruitment and expansion of CD8^+^ T cells inside the tumor was largely the result of STING activation. Further, we observed a significant reduction in Foxp3^+^ regulatory T cell (Treg) populations within tumors (Fig. 1e). In agreement with intra-tumoral elevation in CD8^+^ T cells, DMXAA, alone or with gemcitabine, increased the presence of IFN-γ-producing tumor-reactive CD8^+^ T cells in the spleen (Fig. 1f). Thus, it appears that STING agonist administration after gemcitabine treatment results in systemic anti-tumor immune responses. Taken together, these data suggest STING agonist drives an adaptive T cell tumor-specific immune response that results in pancreatic tumor regression and increased survival.

### STING agonist monotherapy activates anti-tumor immunity and induces regression of pancreatic tumors

**Fig. 2:**
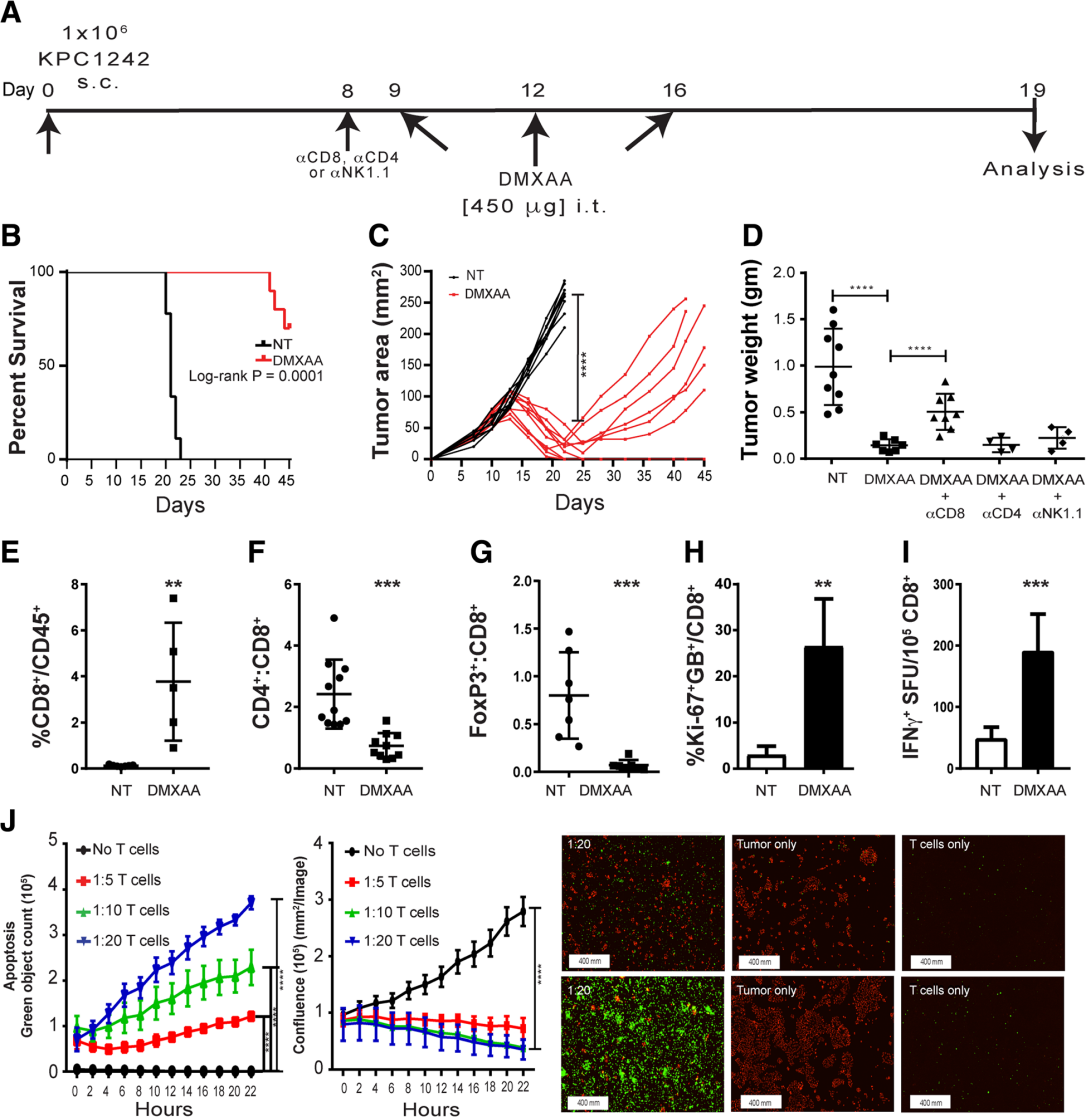
STING activation with DMXAA activates CD8^+^ T cells and induces pancreatic cancer regression in mice. **a** Subcutaneous tumor cell implantation and treatment strategy. **b** Kaplan-Meier survival curves for non-treated (NT) control (black line) and experimental groups. **c** Tumor growth over time in DMXAA-treated (red lines) or control (black lines) mice. Data are from two separate experiments, *n* = 8–10 mice per group. **d** Excised tumor weights were measured 19 days after implantation. CD8, CD4, or NK cells were immunodepleted in vivo and tumor size measured at study end. **e**-**h** Tumors were collected and processed into single cell suspensions and immune profiling of CD8 T cells assessed using flow cytometry. Values are mean ± SD, *n* = 6–8. **i** Spleen-derived CD8^+^ T cells were isolated by immunomagnetic sorting and tested in IFN-γ ELISPOT assays using KPC1242 tumor cells as stimulators. Values are mean IFN-γ spot forming unit (SFU) ± SD. Data are from 1 of 3 replicate experiments, and the CD8^+^ T cells were isolated from the pooled splenocytes of 3 mice. **j** Killing of KPC1242 cancer cells by ex vivo-expanded tumor-infiltrating CD8^+^ T cells from DMXAA-treated tumors. Values are mean ± SD, *n* = 3. Representative images of apoptotic cells (green) and living tumor cells (red) at 2 h (top) and 22 h (bottom). *, *P* ≤ 0.05; **, *P* ≤ 0.01; ***, *P* ≤ 0.001

While dual treatment with the STING agonist and gemcitabine evoked anti-tumor immune responses and provided a strong survival benefit, we noted that DMXAA treatment alone could abrogate tumor progression. We therefore asked whether monotherapy with STING agonist was sufficient to activate the tumor immune microenvironment and ablate tumor formation. We also sought to dissect the immune mechanism(s) regulated by STING agonist treatment in pancreatic cancer. As a first step, we repeated the intra-tumoral treatment of subcutaneously engrafted KPC1242 tumors, increasing the dosing regimen to include an additional treatment with DMXAA 9 days after KPC cancer cell implantation (Fig. 2a). Data in Fig. 2 confirms the powerful anti-tumor effect of STING agonist on tumor formation. Survival of DMXAA-treated mice at day 45 after KPC1242 inoculation was significantly improved as compared to non-treated controls, with 70% of treated mice surviving compared to zero in the non-treated cohort (Fig. 2b). Moreover, in contrast to the 2-injection regimen used in the combination therapy approach outlined in Fig. 1, pancreatic tumor regression was more robust and durable, with 50% of the DMXAA-treated survivors showing complete tumor regression (Fig. 2c). The potent anti-tumor effect of STING agonist monotherapy was confirmed using the KPC1199 cell line as a second pancreatic cancer model. Just as observed with the KPC1242 cell line, three injections of DMXAA nearly abolished KPC1199 tumors (Additional file 1: Figure S1).

Next, we sought to determine the cellular mechanism for the anti-tumor effect. The decreased CD4:CD8 ratio observed in Fig. 1 suggested a role for cytolytic CD8^+^ T cells. Neutralization of CD8^+^ T cells by treatment with an in vivo-depleting monoclonal antibody partially eliminated the anti-tumor effect of DMXAA treatment. In contrast, elimination of CD4^+^ T cells or NK cells with depleting antibodies had no significant effect on tumor size (Fig. 2d). DMXAA treatment of KPC1242 tumors engrafted to RAG-1-deficient recipients had a similar impact on tumor growth as that observed in CD8-depleted wild-type mice (Additional file 2: Figure S2). Confirmation that immunodepletion ablated CD8^+^ T cells or NK cells is shown in Additional file 2: Figure S2. These data suggest cytotoxic T cells have a partial role in the anti-tumor response, and that innate immune cells or non-immune cells may also play a role in DMXAA-mediated anti-tumor effects in PDA.

Consistent with the immune activation observed after DMXAA and gemcitabine combination therapy, tumors harvested from mice treated with 3 intra-tumoral injections of DMXAA alone had increased percentages of CD8^+^ T cells (Fig. 2e, f) and decreased frequencies of Tregs (Fig. 2g) compared to tumor from non-treated mice. Moreover, tumor infiltrating CD8^+^ T cells from STING agonist treated mice were increasingly proliferative and activated, as determined by Ki-67^+^ and granzyme B^+^ expression (Fig. 2h). We also observed a corresponding increase in tumor-reactive CD8^+^ T cells within the spleens of DMXAA-treated mice by IFN-γ ELISPOT assays (Fig. 2i). In addition, when tumor-infiltrating T cells harvested from tumors of DMXAA-treated mice were expanded in culture for 7 days, they were able to effectively kill KPC1242 cells in vitro (Fig. 2j).

**Fig. 3:**
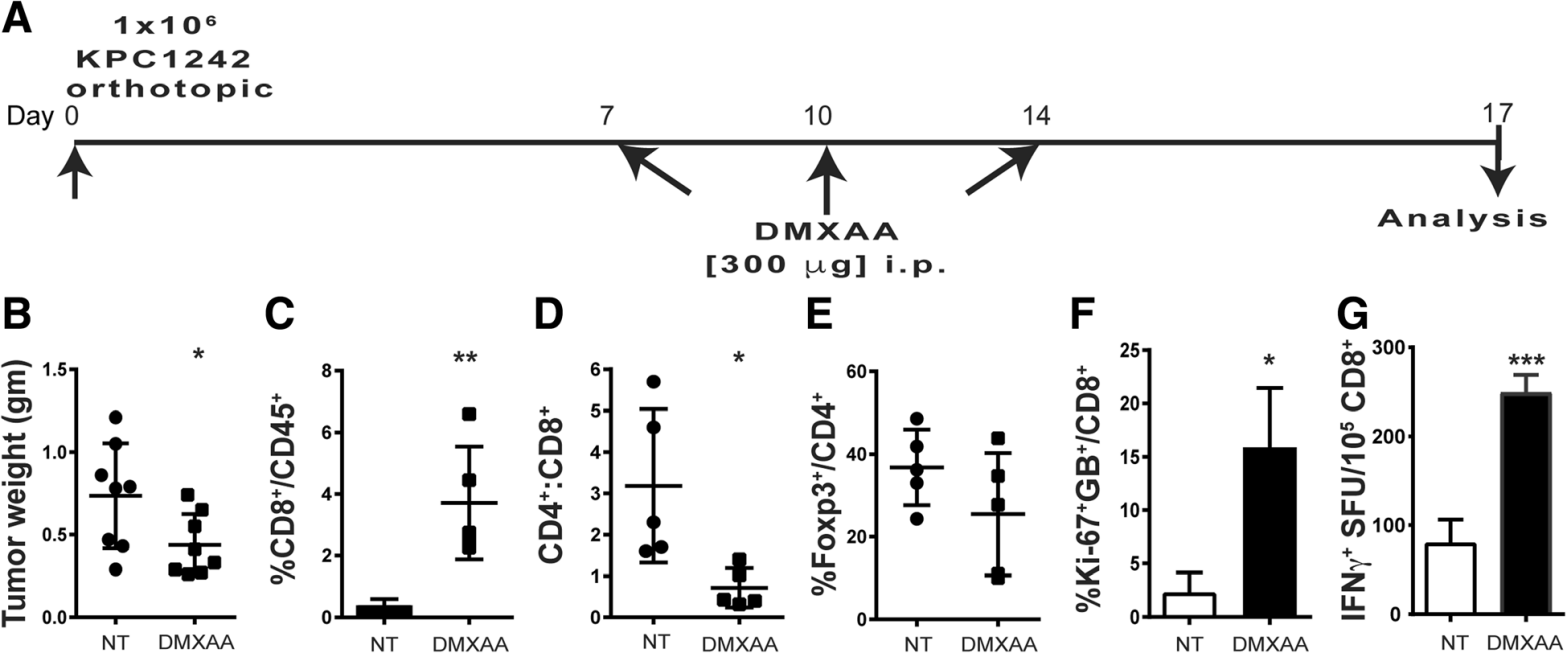
Systemic STING agonist activates CD8^+^ T cells and induces tumor regression in mice with pancreatic cancer. **a** Orthotopic tumor implantation and treatment strategy. **b** Excised tumor wet weights from experimental and control mice. Values are mean ± SD, *n* = 8 mice per group. *, *P* ≤ 0.05. **c**-**e** Tumors were collected and processed into single cell suspensions and immune profiling of infiltrated lymphocytes in DMXAA-treated and control mice completed using flow cytometry. **c** The percentage of CD8^+^ T cells as a percent of tumor infiltrated CD45^+^ cells. **d** CD4:CD8 ratio of CD45^+^ gated leukocytes. **f** Percentage of Foxp3^+^ T cells as a percent of CD4^+^ T cells. **f** Percentage of infiltrating CD8^+^ T cells expressing Ki-67 and Granzyme B (GB) within the tumor. **g** Spleen CD8^+^ T cells were isolated by immunomagnetic sorting and tested in IFN-γ ELISPOT assays using KPC1242 tumor cells as stimulators. Graphs represent the mean spot forming units (SFU) ± SD of 2 independent experiments. *, *P* ≤ 0.05; **, *P* ≤ 0.01; ***, *P* ≤ 0.001

Next, we sought to determine if systemic STING agonist therapy would promote anti-tumor immune responses to mice with orthotopic pancreatic tumors. KPC1242 cells were implanted to the pancreas following our well-established protocol [41–44], and DMXAA was administered systemically via intraperitoneal injection. To minimize potential innate immune hypersensitivity or cytokine storm, we lowered the dose of DMXAA to 300 μg per injection and repeated the monotherapy approach, injecting drug on days 7, 10, and 14 after implantation (Fig. 3a). As shown in Fig. 3b, systemic administration of DMXAA provided a modest but significant reduction in the size of orthotopically implanted tumors, as measured by tumor wet weight 17 days after implantation. Consistent with data from intra-tumoral injected pancreas tumors, intraperitoneal administration of DMXAA triggered a significant infiltration and accumulation of CD8^+^ T cells within PDA tumors (Fig. 3c), with a concomitant reduction in the CD4:CD8 T cell ratio (Fig. 3d). Similar to our findings in DMXAA-treated subcutaneous tumors, we observed decreased percentages of Foxp3^+^ Treg cells in the orthotopic tumors of mice treated systemically with DMXAA (Fig. 3e). The proliferation and activation of functional orthotopic tumor-infiltrating CD8^+^ T cells was monitored by staining with Ki-67 and granzyme B. Much as we observed in subcutaneous tumors, there was a significant increase in granzyme B^+^ and Ki-67^+^ CD8^+^ T cells within the orthotopic pancreatic tumors after DMXAA treatment (Fig. 3f). Not surprisingly, higher numbers of CD8^+^ T cells in the spleens of DMXAA-treated mice produced IFN-γ in response to KPC1242 cells in vitro (Fig. 3g). Consistent with the literature, there were relatively few CD8^+^ T cells detected inside non-treated orthotopic KPC1242 tumors [51–53]. Not only were CD8^+^ T cells scarce within control tumors, they were functionally deficient based on a lack of granzyme B or Ki-67 expression (Fig. 3f). These results suggest that systemic treatment with STING agonist reduced tumor size and potently increased the infiltration and functional activation of tumor-reactive cytolytic T cells.

### DMXAA stimulates proinflammatory cytokine and chemokine production in vivo

**Fig. 4:**
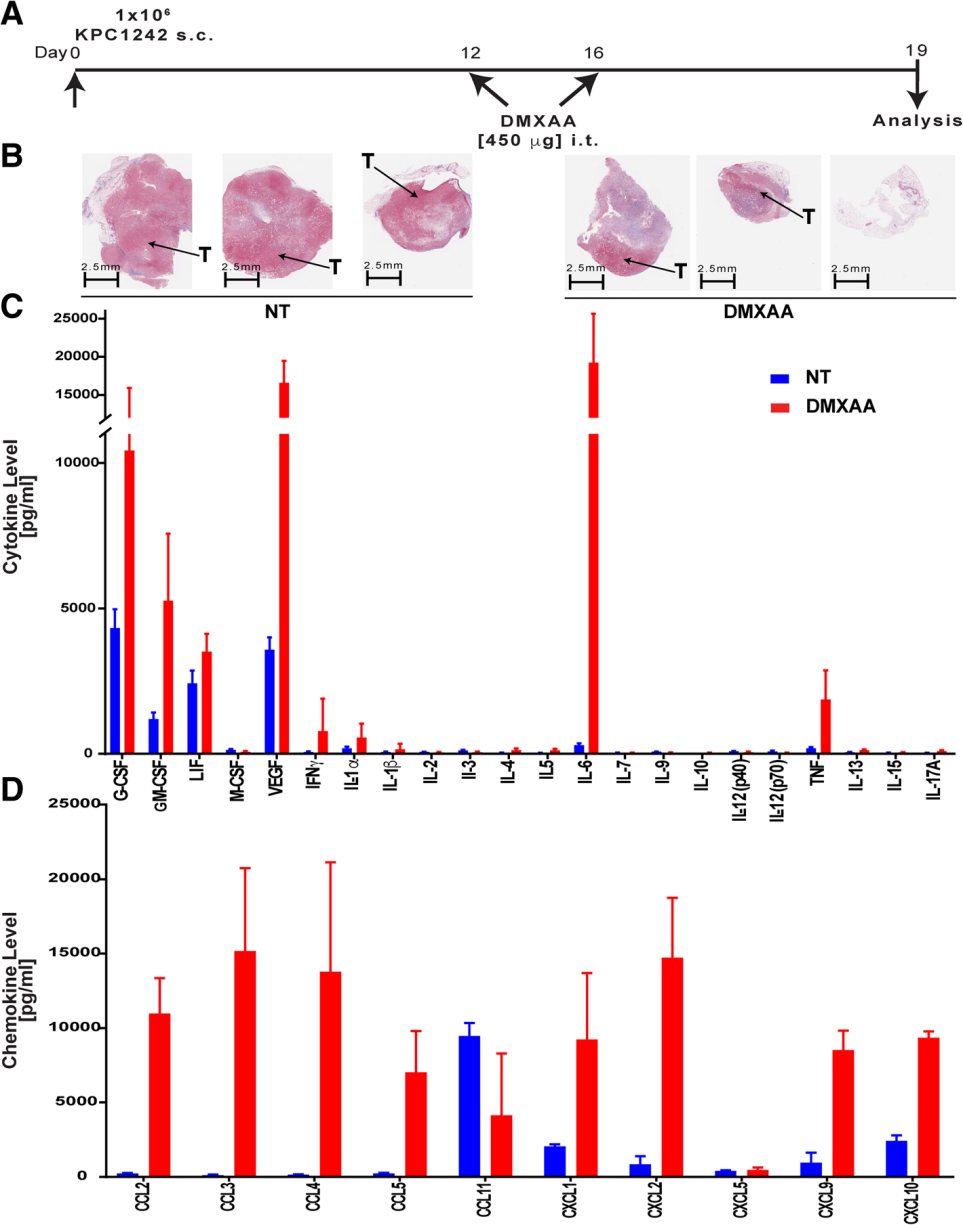
DMXAA induces the production of proinflammatory cytokines and chemokines in vivo. **a** Timeline for intra-tumoral treatment with DMXAA. Control mice were not treated (NT). **b** Representative histopathologic tissue specimens stained with Masson’s trichrome from untreated and DMXAA-treated mice. Tumor tissues (T) stain red while collagen stains blue. **c**, **d** Multiplex analysis of intra-tumoral cytokines and chemokines in non-treated (NT, blue bars) or DMXAA-treated (red bars) tumors. Values are mean ± SD, *n* = 9 control non-treated and 15 DMXAA treated mice from 2 independent experiments

Our data from PDA engrafted immune competent mice treated with CD8 neutralizing antibody or engrafted to Rag-deficient mice suggested that the anti-tumor effects of STING agonist required both CD8^+^ T cells as well as accessory innate cells. To rigorously establish a role for STING agonist in T cell accumulation and activation within the previously suppressed pancreatic tumors, a 31-plex bead-based assay was performed to quantify levels of inflammatory mediators within the pancreatic tumor microenvironment on day 19, 3 days after a second DMXAA injection (Fig. 4a). As illustrated in Masson trichrome stained tissue sections, DMXAA -treated PDA tumors were smaller, than untreated controls, with fewer red-staining tumor epithelial cells (Fig. 4b). Moreover, several critical inflammatory cytokines including TNFα, IFN-γ, IL-1α, and IL-6 were markedly increased within DMXAA-treated pancreatic tumors compared to control tumors (Fig. 4c). This inflammatory phenomena within the tumor microenvironment is indicative of a potent and broad immune activation within the tumor microenvironment and agrees with the observed activation levels of tumor-infiltrating T cells shown in Figs. 2 and 3 [54, 55]. There was also a significant increase in growth factors G-CSF, GM-CSF, and LIF within the PDA tumors treated with DMXAA compared to controls (Fig. 4c). In addition to those inflammatory cytokines, DMXAA treatment significantly increased intra-tumoral levels of the chemokines CCL2, CCL3, CCL4, CCL5, CXCL1, CXCL2, CXCL9 and CXCL10 (Fig. 4d). These chemokines play roles in regulating the migration of subsets of innate and adaptive immune cells including monocytes, macrophages and T cells. Interestingly, CCL11, an eosinophil chemoattractant typically associated with Th2 immune responses, was decreased in DMXAA-treated tumors. Taken together, these unbiased multiplex data from in vivo tumors indicate that STING agonist treatment potently inflames the previously suppressed pancreatic cancer microenvironment, resulting in decreased tumor size due to the increased inflammatory response.

### DMXAA reprograms TAMs in vivo and induces macrophage activation in vitro

**Fig. 5:**
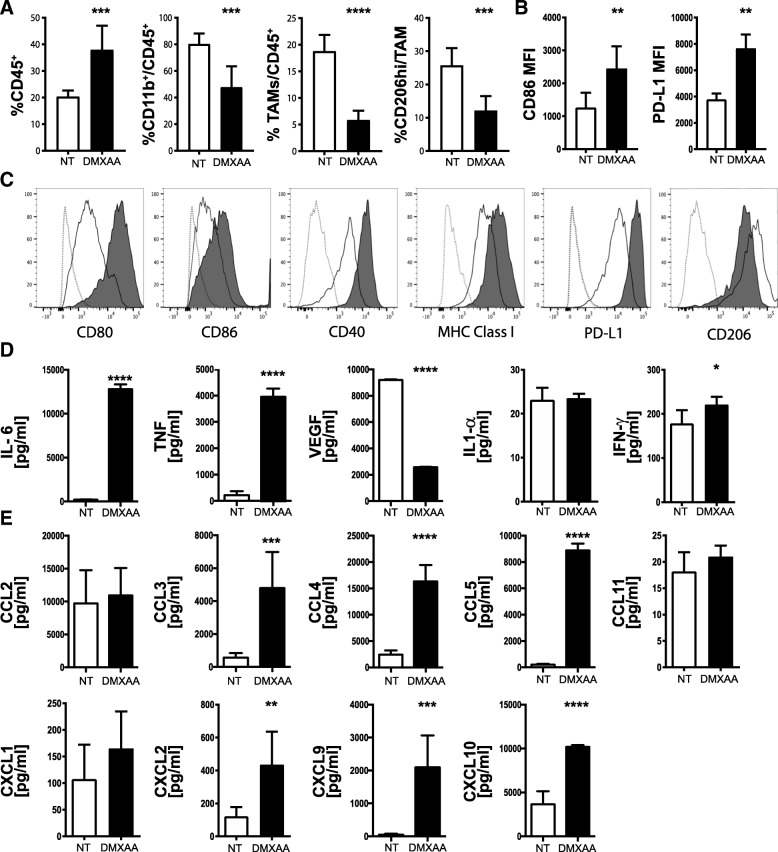
DMXAA reprograms TAMs in vivo and activates macrophages in vitro. Tumors were harvested on day 19, dissociated and immune cell subsets analyzed by flow cytometry. **a** Percent of viable CD45^+^ leukocytes, CD11b^+^,Ly6G^−^, Ly6C^Lo^, F4/80^Hi^, MHC Class II^+^ myeloid tumor-associated macrophages (TAM), and CD206^hi^ TAMs. **b** Mean fluorescence intensity (MFI) of CD86 and PD-L1 levels expressed on CD45^+^CD11b^+^ TAMs from control (NT, open bar) or DMXAA [450 μg] (black bar) treated mice. Values are mean ± SD, n = 6 mice per group. *, *P* ≤ 0.05; **, *P* ≤ 0.01. **c** Cultured bone marrow-derived macrophages were untreated or treated for 18 h with 20 μg/ml DMXAA. Histograms from representative flow cytometry analyses for CD80, CD86, CD40, MHC class I, PD-L1 and CD206 expression on bone marrow-derived macrophages from non-treated (black line) DMXAA-treated (black area) and compared against unstained cells (gray dotted lines). **d**, **e** Multiplex analyses of cytokine and chemokine production measured in conditioned medium from bone marrow-derived macrophages cultured 18 h in the presence (black bars) or absence (open bars) of 20 μg/mL DMXAA. Values are mean ± SD of two combined experiments. *, *P* ≤ 0.05; **, *P* ≤ 0.01; ***, *P* ≤ 0.001

Tumor-associated macrophages (TAMs) in the pancreatic cancer microenvironment are known to promote cancer cell proliferation, angiogenesis, and mediate immunosuppression to support tumor growth and metastasis [56]. Further, reprogramming immunosuppressive TAMs in the tumor microenvironment has been shown to augment the beneficial effects of immunotherapy [57]. To determine the impact of STING signaling on myeloid responses in pancreatic cancer, we profiled the composition and phenotype of tumor-infiltrating myeloid cells. Using the same treatment schedule depicted in Fig. 4a, isolation and flow cytometric enumeration revealed a significant increase in intra-tumoral CD45^+^ leukocytes 3 days after the third injection of DMXAA (Fig. 5a). The percent of total CD11b^+^ myeloid cells and TAMS characterized as CD11b^+^, Ly6G^−^, Ly6C^Lo^, F4/80^Hi^, and MHC class II^+^, was significantly decreased in DMXAA-treated tumors (Fig. 5a). CD206^Hi^ TAMs, known to be immunosuppressive M2-type, also decreased in frequency. Of the TAMs present in DMXAA-treated tumors, surface expression of the CD86 co-stimulatory molecule was increased (Fig. 5b). There was also an elevation in surface levels of the co-inhibitory molecule PD-L1 on TAMs from DMXAA-treated tumors. The elevated PD-L1 expression was likely due to the increased production of IFN-γ by immune cells in the tumor microenvironment after STING agonist administration.

Bone marrow-derived macrophages (Additional file 3: Figure S3) were treated with DMXAA in vitro to further investigate its potential role on immune activation within pancreatic tumors. In parallel with the increased expression of CD86 observed in vivo, we determined that multiple co-stimulatory molecules involved in T cell activation including CD80, CD86 and CD40, and MHC Class I were upregulated by DMXAA treatment in culture (Fig. 5c). CD206 expression on DMXAA-treated macrophages was decreased both in vivo (Fig. 5a) and in vitro (Fig. 5c). As TAMs are predominantly M2-macrophages [58] we tested the effect of DMXAA on M2-polarzied macrophages in vitro. In agreement with our in vivo findings from tumor-isolated TAMs, DMXAA efficiently activates M2-polarized macrophages, resulting in the highest upregulation of MHC and costimulatory molecules compared to LPS-activated M1-type macrophages and DMXAA single treated bone marrow-derived macrophages (Additional file 3: Figure S3). In vitro*,* DMXAA-treated macrophages produced increased levels of IL-6, TNFα, and to an extent IFN-γ (Fig. 5d). In contrast to the whole tumor levels observed in vivo, VEGF was decreased in cultured DMXAA-treated macrophages. Further, several chemokines including CCL3, CCL4, CCL5, CXCL2, CXCL9, and CXCL10 were secreted by STING activated macrophages (Fig. 5e). Together, these data suggest that intra-tumoral DMXAA treatment of KPC1242 tumors repolarizes suppressive M2-type macrophages to an inflammatory M1-type within the tumor microenvironment, which likely plays a role in promoting the recruitment and activation of cytotoxic T cells.

### STING agonist monotherapy induces dendritic cell activation and maturation in vivo and in vitro

**Fig. 6:**
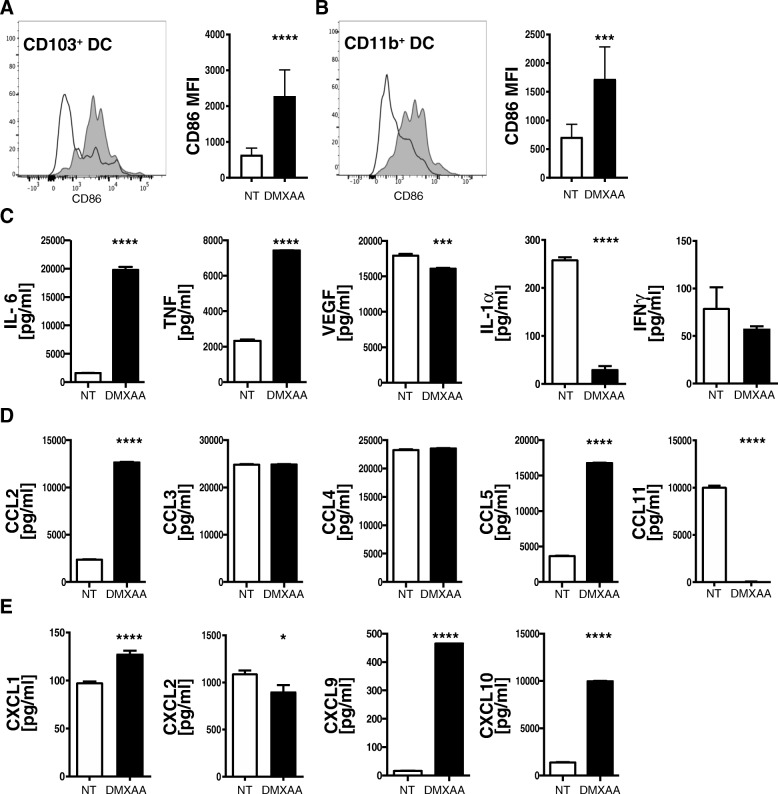
STING agonist induces DC activation and maturation in vivo and in vitro. Tumors were harvested on day 19, digested and viable CD45^+^ immune cell subsets analyzed by flow cytometry. Co-stimulatory molecule CD86 expression on tumor-infiltrating MHC Class I expressing CD103^+^ (**a**) or CD11b^+^ (**b**) DC in non-treated controls (black lines or open bars) or DMXAA treated tumors (shaded gray histograms or black bars). Values are mean ± SD, n = 8–9 mice per group. ***, *P* ≤ 0.001; ****, *P* ≤ 0.0001. Cytokines (**c**), CC-family chemokines (**d**) or CXC-family chemokines (**e**) were measured in conditioned medium from bone marrow-derived DCs cultured 18 h in the presence (black bars) or absence (open bars) of 20 μg/mL DMXAA. Data are representative of two experiments. *, *P* ≤ 0.05; **, *P* ≤ 0.01; ***, *P* ≤ 0.001; **** *P* ≤ 0.0001

T cells are dependent upon professional antigen presenting cells, such as dendritic cells (DC), for their activation in response to cognate antigens. The ability of DCs to induce a T cell response depends on DCs activation and maturation status. Based on the increased frequencies of tumor-reactive T cells in DMXAA-treated tumors and data in the literature documenting the importance of DCs to STING-driven anti-tumor responses [36, 59], we hypothesized that DCs in the DMXAA-treated PDA microenvironment are mature and play a role in the accumulation of T cells. To monitor the activation status of DCs in KPC1242 tumors, the expression of CD86 on CD103^+^ or CD11b^+^ subsets of tumor resident MHC Class II^+^, CD11c^+^, Ly6C^−^, and CD64^−^ DCs was quantified. Consistent with the tumor being immune suppressed, both CD103^+^ DCs and CD11b^+^ DCs in non-treated tumors expressed relatively low levels of the activation marker CD86 (Fig. 6a, b). While DMXAA treatment did not significantly increase the frequency of CD103^+^ DCs within the tumor (data not shown), there was a profound and significant increase in CD86 expression on both CD103^+^ DCs and CD11b^+^ DCs in tumors treated with STING agonist.

Congruent with those in vivo data, DMXAA stimulation of bone marrow-derived DCs in vitro generated significantly higher amounts of the proinflammatory cytokines IL-6 and TNFα (Fig. 6c), as well as increased levels of the chemokines CCL2, CCL5, CXCL1, CXCL9 and CXCL10 (Fig. 6d, e). Notably, with the exception of VEGF, each of the chemokines and cytokines that were elevated in DMXAA-treated bone marrow-derived DCs paralleled cytokines shown to be elevated within DMXAA-treated tumors in vivo (Fig. 4c, d). Notable differences with DMXAA-treated bone marrow-derived macrophages and DCs were detected in levels of CCL2, CCL3 and CCL4. Macrophages produced increased amounts of CCL3 and CCL4 while DCs preferentially secreted CCL2. These data suggest that DCs in the tumor microenvironment contribute to production of immune stimulatory factors in response to DMXAA. Further, these data support the notion that DCs within the untreated pancreas tumor are ‘immature’ and poorly stimulatory to T cells prior to their activation and differentiation into mature antigen cross-presenting DCs after STING agonist treatment.

### Pancreatic cancer epithelial cells upregulate proinflammatory genes after STING agonist treatment

**Fig. 7:**
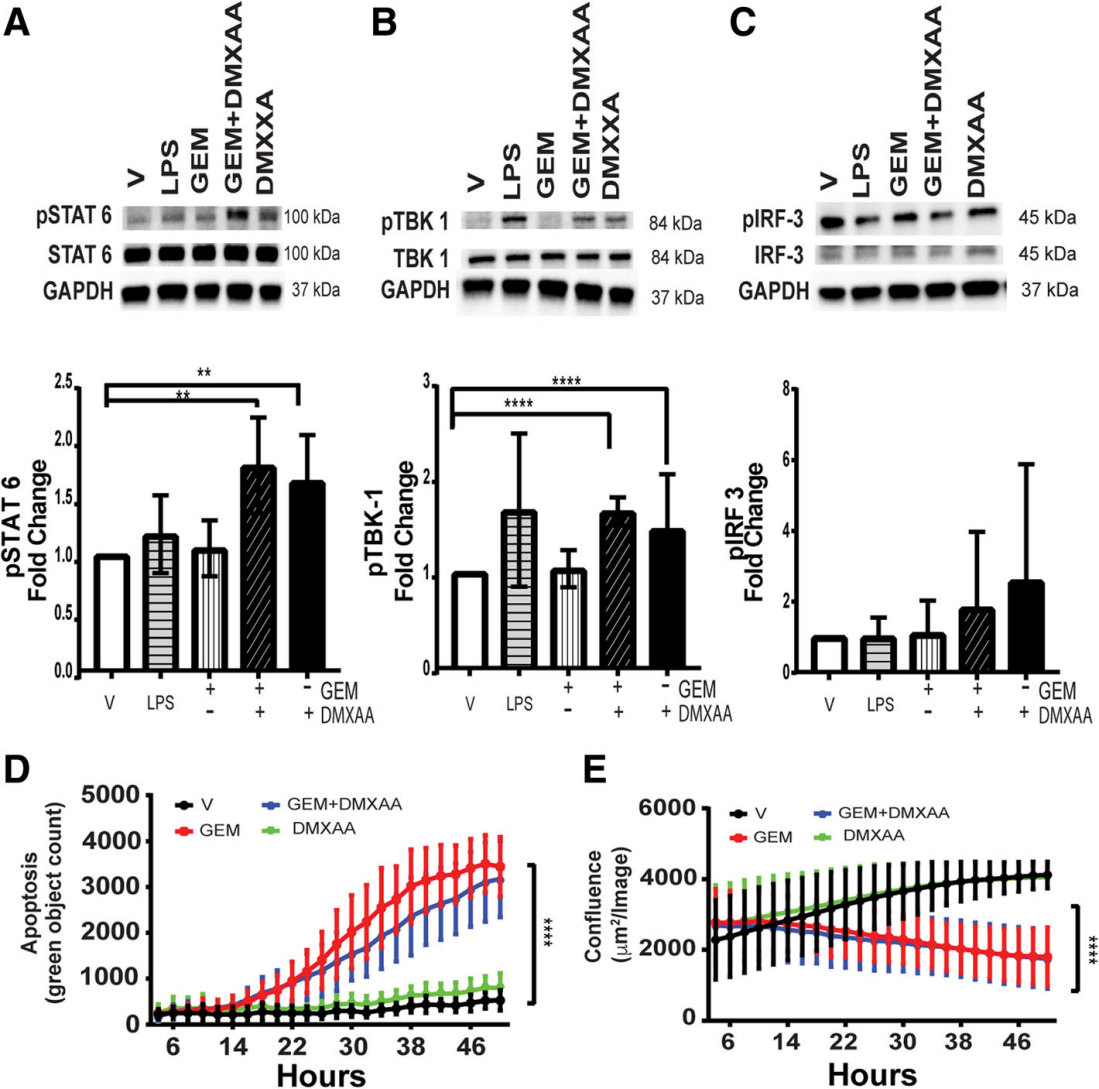
Pancreatic cancer epithelial cells are activated by STING agonist. **a** KPC1242 cells were stimulated with 10 μg/mL gemcitabine (GEM) alone, with GEM with 100 μg/mL DMXAA, DMXAA alone, 1 μg/mL lipopolysaccharide (LPS) as a positive control, or a vehicle negative control. Protein lysates were analyzed with antibodies to (**a**) phospho-STAT6 (pSTAT6), total STAT6 or GAPDH. **b** phospho-TBK1, total TBK1, or GAPDH, (**c**) phosphoIRF-3, total IRF-3 or GAPDH. Blots were probed with antibody against GAPDH as a loading control. Immunoblots were quantitated and represented graphically below each respective blot. **d** KPC1242 cells were incubated with GEM alone, GEM plus DMXAA, DMXAA alone, LPS alone, or vehicle control and apoptosis (d) and cell growth (e) measured. **, *P* ≤ 0.01; ****, *P* ≤ 0.0001. Values are mean ± SD, *n* = 4

While the partial response of KPC1242 tumors treated with DMXAA likely reflects its impact on lymphocytes and leukocytes, it is entirely plausible that cancer epithelial cells directly respond to STING agonist and participate in re-activation of the immune suppressed tumor. It is well established that epithelial cells are potent activators of proinflammatory signaling in infectious diseases and cancer [60–66]. Roles for epithelial cancer cells in immune suppression of malignant tumors or active inflammation in an immunologically ‘hot’ tumor microenvironment remains little understood. While pancreatic cancer cells may make up only a fraction of the overall tumor mass, epithelial cells would have an outsize role in re-activating and recruiting T cells to infiltrate the tumor parenchyma. We first asked if DMXAA is recognized by KPC cells in culture and could, in turn, activate proinflammatory signaling pathways. As shown in Fig. 7a-c, treatment of KPC1242 cancer cells with DMXAA, alone or in combination with gemcitabine, increased phosphorylation of TBK1 and STAT6, two cardinal STING signaling pathways. Levels of IRF3 were more variable but similarly trended to increased phosphorylation. Further, STING agonist had little to no impact on cancer epithelial cell proliferation or apoptosis (Fig. 7d, e). These data indicate that pancreatic cancer epithelial cells are targets of DMXAA and that the beneficial effects on tumor regression are independent of tumor autonomous effects on cellular growth or death.

**Fig. 8:**
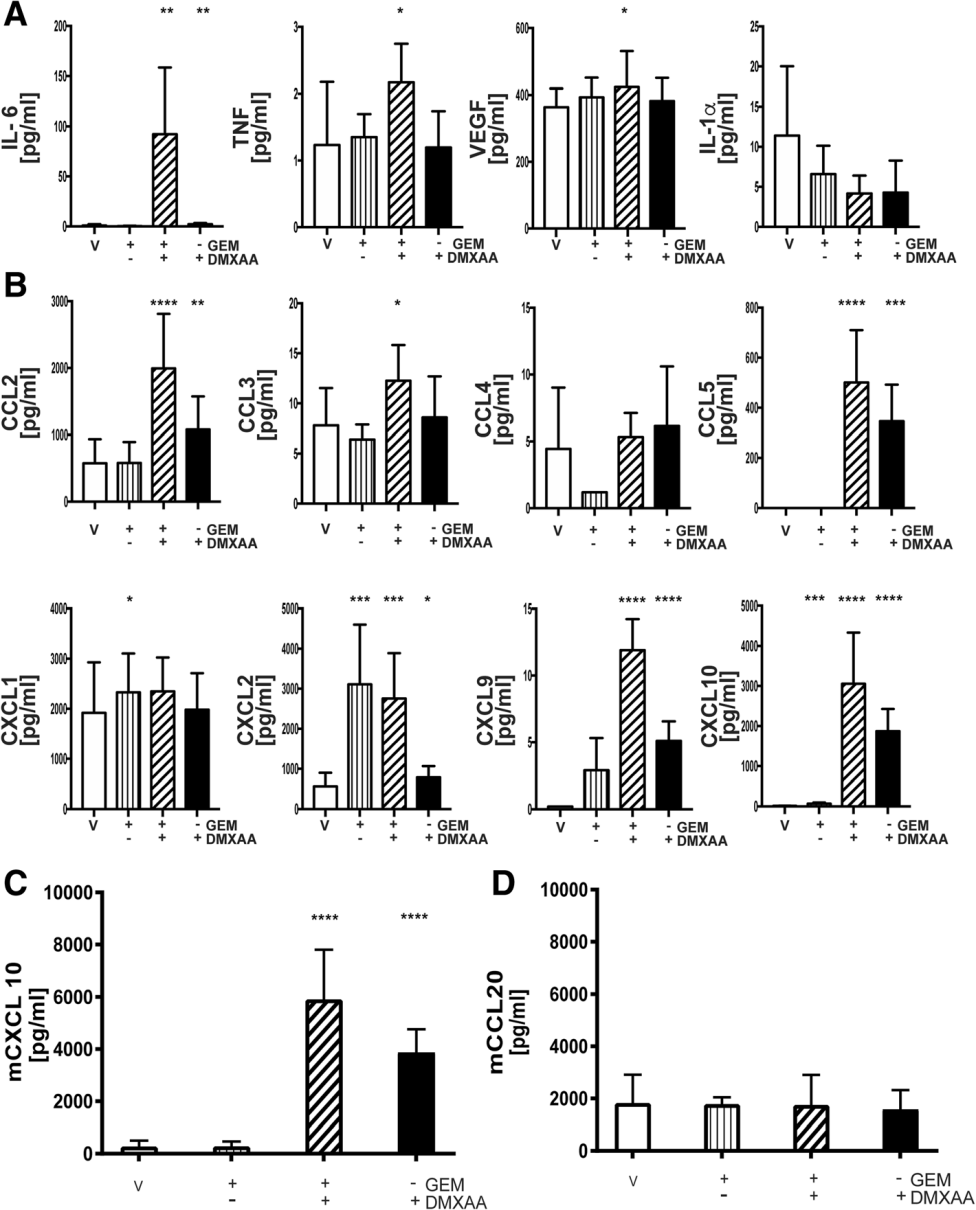
STING agonist stimulates T cell chemokine expression. Conditioned medium from KPC1242 cells that had been stimulated with GEM alone, 10 μg/mL GEM plus 100 μg/mL DMXAA, DMXAA alone, or vehicle as a control were analyzed by MultiPlex or ELISA. Cytokine **a** and chemokine (**b**) levels in conditioned medium from treated and control cells. Data are triplicate means from a representative of 3 independent analyses. **c**, **d** CXCL10 and CCL20 chemokine levels measured by ELISA. Data are mean ± SD, *n* = 4 biological replicates completed in triplicate. ****, *P* ≤ 0.0001

A multiplex cytokine/chemokine array was next used as an unbiased screen to determine if DMXAA stimulation modulated the production of proinflammatory cytokines and/or chemokines by pancreatic cancer epithelial cells. Data in Fig. 8a suggest DMXAA stimulated the robust production of IL6. In contrast to in vivo or in vitro DMXAA-treated DCs and macrophages, pancreatic cancer epithelial cells produced little if any TNFα, VEGF or IL-1α. Moreover, there was elevated secretion of T cell attractant chemokines CCL5, CXCL9 and CXCL10, monocyte/macrophage attractants, CCL2, CCL3, and CCL4, as well as the neutrophil chemokines, CXCL1 and CXCL2 (Fig. 8b).

Conventional wisdom is that STING mediates its proinflammatory effects and anti-tumor T cell responses by activating DCs. Using ELISA, we asked if pancreatic cancer cells produced the predominant mucosal DC-specific chemokine CCL20. As shown in Fig. 8c, d, STING agonist upregulated the T cell chemokine CXCL10 with little effect on the DC attractant CCL20, suggesting that STING agonist therapy increases the adaptive anti-tumor T cell responses through multiple cellular mechanisms. Cumulatively, these data support the notion that DMXAA reignites the immunologically suppressed pancreatic cancer tumor. This effect reflects the broad upregulation of proinflammatory cytokines and chemokines which likely elevate the level of tumor-reactive T cells and reprogram TAMs and DCs into anti-tumor inflammatory subsets.

## Discussion

Recent evidence showing that activation of the innate immune system is a viable anti-cancer therapeutic approach prompted us to test the hypothesis that treatment of PDA-bearing mice with murine STING agonist, DMXAA, would induce tumor regression. We used cancer cell lines isolated from autochthonous KPC mouse tumors expressing the overactive mutant allele of KRas and a dominant negative allele of Trp53 characteristic of most human pancreatic cancers [47, 67]. The KPC transgenic mouse represents a physiologically relevant model that phenocopies several key aspects of human pancreatic cancer, including immune suppression and desmoplasia [67]. We employed subcutaneous and orthotopic in vivo KPC implantation approaches to demonstrate potent roles for STING agonists in modulating the immune microenvironment of pancreatic cancer. We found that DMXAA treatment, either by intra-tumoral injections into subcutaneous tumors or by intraperitoneal injections to orthotopic tumors, induced a significant survival advantage and reduction in tumor burden as compared to non-treated animals. Additionally, we determined that activation of the STING pathway increases the frequency and fitness of cytotoxic T lymphocytes and decreases the frequency of regulatory T cells and suppressive macrophages within the tumor. STING agonist administration also increased intra-tumoral secretion of inflammatory cytokines and T cell-attracting chemokines, thereby reinvigorating a potent anti-tumor immune response within the tumor mass. Furthermore, specific anti-tumor effector T cell activity was observed in the periphery of DMXAA-treated mice, suggesting intra-tumoral injection of STING agonist can modulate systemic anti-tumor immunity. This observation is especially relevant in the context of metastatic disease. These results demonstrate the potential utility of STING agonists as anti-cancer therapeutics for non-immunogenic tumors such as PDA.

Only two other recent reports have highlighted STING agonist treatment of pancreatic tumors [68, 69]. Most recently, a separate KPC cell line from those used here were embedded within biopolymer scaffolds containing the STING agonist cdGMP followed by adoptive T cell transfer of NKG2D-specific chimeric antigen receptor (CAR)-modified T cells. While treatment with STING agonist alone modestly extended survival time (i.e., all mice still died from tumor progression), combined treatment with CAR-T cells resulted in a dramatic synergistic improvement on overall survival [69]. While these data suggest a role for STING agonists as adjuvants, the mechanisms for this beneficial effect was limited to documenting increased numbers of activated DCs following treatment. The potential curative effects of synthetic STING ligands were described in a separate report using the murine chemical-induced Panc02 tumor model [68]. In this report, intra-tumoral injection with STING ligand resulted in elevated levels of TNFα, IL6 and CCL2, but no significant increase in IFNγ within the Panc02 tumor microenvironment. In contrast to those reports, we found STING agonist treatment of the more physiologically relevant KPC1242 and KPC1199 tumors produced a sustained elevation in TNFα, IL6, CCL2 and, notably, IFNγ. This highlights the likely importance of these cytokines both early and later in the anti-tumor response. Moreover, our analysis went further and documented increases in additional cytokines and chemokines and determined the individual cell subsets responsible for the STING-induced elevation in cytokine and chemokine levels. Notably, the T cell chemokine attractants CCL5, CXCL9 and CXCL10 were increased in DMXAA-treated tumors, macrophages and DCs, as well as KPC1242 epithelial tumor cells, suggesting that these chemokines could participate in the recruitment of T cells to STING agonist-treated pancreatic tumors. In future studies, we will address the role of these chemokines in facilitating generation of the increased anti-tumor T cell response induced by STING agonist.

In agreement with a prior report in STING agonist-treated melanomas [36], we determined that administration of DMXAA provided a marked, albeit partial, therapeutic benefit in T cell or NK cell deficient mice, suggesting that other innate immune cells and/or non-immune cells play key roles in the anti-tumor responses induced by STING activation. While the majority of current analyses have focused on activation of STING in DCs, the presence of STING in non-immune cells and cancer epithelial cells also may participate in promoting efficacy of STING agonists. Further, differential expression of STING in cancer cells and stromal support cells, as well as differential levels of immune suppression suggest that STING agonist effects are likely variable and tumor-dependent. For instance, deletion of STING in B16D8 melanoma cells has been demonstrated to have minimal impact on cell survival [70], and administration of STING agonists did not affect cell growth of B16F10, SCCFVII, or CT26 cells [71, 72]. In contrast, breast cancer cell lines 4 T1, MCF-7, and T47D appear sensitive to changes in STING expression or administration of STING agonists [73, 74]. Ongoing work will evaluate the expression of STING and sensitivity to STING agonists in various human and mouse pancreatic cell lines.

The use of STING agonists as therapeutic agents in pancreatic cancer has the potential to greatly improve strategies that target the tumor microenvironment. A major feature of pancreatic adenocarcinoma is the generation of a desmoplastic tumor histology that renders neoplastic cells insensitive to standard treatments such as chemotherapy or immunotherapy [75]. In contrast to robust activity observed in the use of single agent checkpoint inhibitors targeting CTLA-4 or PD-1 in many other cancers, activity in pancreatic cancer has been minimal [14, 21], likely resulting from the immunologic inert microenvironment and limited T cell infiltration of the tumor parenchyma [20]. Our studies indicate that STING agonists can enhance the presence of effector CD8^+^ T cells within pancreatic cancer. Moreover, we reported that this infiltration could be stimulated by systemic STING agonist, suggesting non-tumoral injection approaches may have clinical utility. In agreement, a recent report demonstrated that STING agonists induce inflammation of the pancreas in an experimental model of acute pancreatitis, with systemic DMXAA increasing leukocyte recruitment into the tissues of the inflamed pancreas [76]. Our data therefore indicate that STING agonists fundamentally alter the pancreatic tumor environment in ways that improve immune accessibility. Consistent with this notion, we showed that intra-tumoral CD103^+^ DCs, known to play a role in cross-presentation of tumor antigen, expressed increased levels of the dendritic cell activation marker and T cell co-stimulatory ligand CD86.

Initial clinical trials evaluating STING agonists were unsuccessful because DMXAA, the first compound tested in human patients, efficiently binds mouse STING, but is unable to bind human STING [77]. The discovery of discrepant STING binding affinities between the two species led to development of additional STING agonists that were capable of binding both human and mouse STING [36]. While STING is capable of changing the immunologic landscape of the tumor microenvironment, it is clear from phase I clinical trials that optimization of the therapy will require concurrent activation of T cells, such as through antibodies that block anti-PD1 or anti-PD-L1. In fact, preclinical data suggest that anti-PD1 efficacy is dependent on the presence of STING [78]. Multiple current trials are exploring the safety and efficacy of combination STING/anti-PD1/PD-L1 approaches. Gemcitabine, alone or in combination with nab-paclitaxel is the standard of care therapeutic options for resectable and advanced pancreatic cancer patients [79]. Gemcitabine is a nucleoside analog used to block DNA replication and evoke apoptosis of cancer cells in vitro and in vivo [79, 80]. We speculate that the additive effects we noted for DMXAA and gemcitabine may reflect T cell-mediated killing in combination with chemotherapy-induced cell death. Alternatively, gemcitabine may release neo-antigens that are recognized by the newly activated and infiltrating T cells. A caveat to using gemcitabine with immune-based therapies is the potential for it to suppress bone marrow functions and evoke leukopenia [81]. This caveat may be mitigated by STING agonist treatment as we noted a strong upregulation of myeloid and granulocyte lineage growth factors in treated pancreatic tumors.

In summary, we have demonstrated a powerful role for an innate sensory signaling pathway in the reactivation of the immunologically suppressed microenvironment of pancreatic tumors. This response showed breadth across immune and non-immune cell types within the tumor microenvironment. Given the breadth of STING agonists available, our data provide rationale for clinical trials to test the safety and efficacy of STING agonists. Our data on the potential additive effects with cytotoxic therapies such as gemcitabine suggest that STING agonists should additionally be tested in combination with tumor-damaging radiation or standard-of-care chemotherapeutic agents to improve the treatment efficacy of patients with pancreatic adenocarcinoma.

## Additional files

Additional file 1: Anti-tumor effects of STING agonist on KPC1199 pancreatic tumors. One million KPC1199 pancreatic cancer cells were inoculated to the subcutaneous dorsal flank and allowed to grow. Mice were treated either with vehicle (PBS) or with three intra-tumoral injections of 450 μg in a 50 μL volume of DMXAA. STING agonist was administered on day 12, 16, and 18 post implantation. Tumor wet weight (left) and area (right) on day 20 indicated robust decrease in tumor progression in mice treated with DMXAA. *n* = 3 mice per group. (PDF 355 kb)

Additional file 2: Anti-tumor effects of STING agonist on αCD8/αNK1.1 pancreatic tumors. (A, G) Experimental treatment strategy of subcutaneous pancreatic tumors in C57BL/6 mice. Control (NT) or experimental mice were treated as indicated (B) Pancreatic tumor wet weights were measured from Rag-1 deficient mice that had been implanted with KPC1242 cells and left not treated as a control or treated with DMXAA (C). Tumors were collected 19 days after implantation and tumor weight measured. (D-F) Tumors were processed into single cell suspensions and CD4:CD8 ratios and percent CD8^+^ and CD4^+^cells within the CD45^+^ compartment determined by flow cytometry. *n* = 4 mice per group (H). Kaplan-Meier survival curves are shown for the indicated control and experimental groups. *n* = 7 mice per group. (I) Tumor growth over time in mice DMXAA-treated (red lines), DMXAA + αCD8/NK1.1 treated (blue lines) or NT control (black lines). *, *P* ≤ 0.05; **, *P* ≤ 0.01; ***, *P* ≤ 0.001; ****, *P* ≤ 0.0001. (TIF 582 kb)

Additional file 3: Bone marrow-derived macrophage characterization. Isolated bone marrow–derived macrophages were cultured in G-CSF alone (M0) or polarize activated on day 7 by change fresh medium containing either 100 ng/ml LPS, to simulate M1 activation, or 10 ng/ml IL4 to induce M2 polarization for 48 h. Some M2 activated BMDMs were stimulated with 20 μg/mL DMXAA for 18 h and stained for flow cytometry analyses. (TIF 262 kb)
