# Supplementary figures and images for: STING agonist inflames the pancreatic cancer immune microenvironment and reduces tumor burden in mouse models
Source: J Immunother Cancer. 2019 Apr 29;7:115. doi: 10.1186/s40425-019-0573-5 (PMC6489306; doi:10.1186/s40425-019-0573-5)

**A**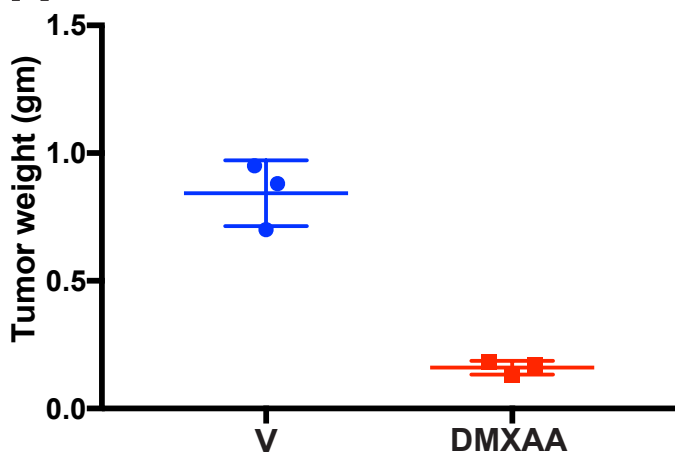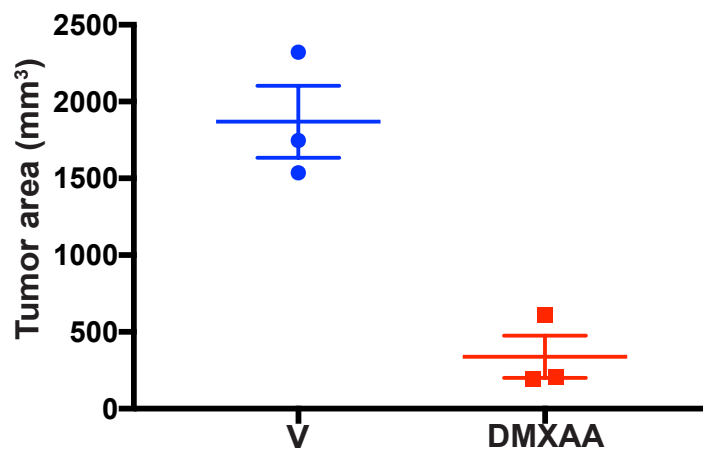

Supplement: Supplementary file 1 — Anti-tumor effects of STING agonist on KPC1199 pancreatic tumors. One million KPC1199 pancreatic cancer cells were inoculated to the subcutaneous dorsal flank and allowed to grow. Mice were treated either with vehicle (PBS) or with three intra-tumoral injections of 450 μg in a 50 μL volume of DMXAA. STING agonist was administered on day 12, 16, and 18 post implantation. Tumor wet weight (left) and area (right) on day 20 indicated robust decrease in tumor progression in mice treated with DMXAA. n = 3 mice per group. (PDF 355 kb) [file 40425_2019_573_MOESM1_ESM.pdf]

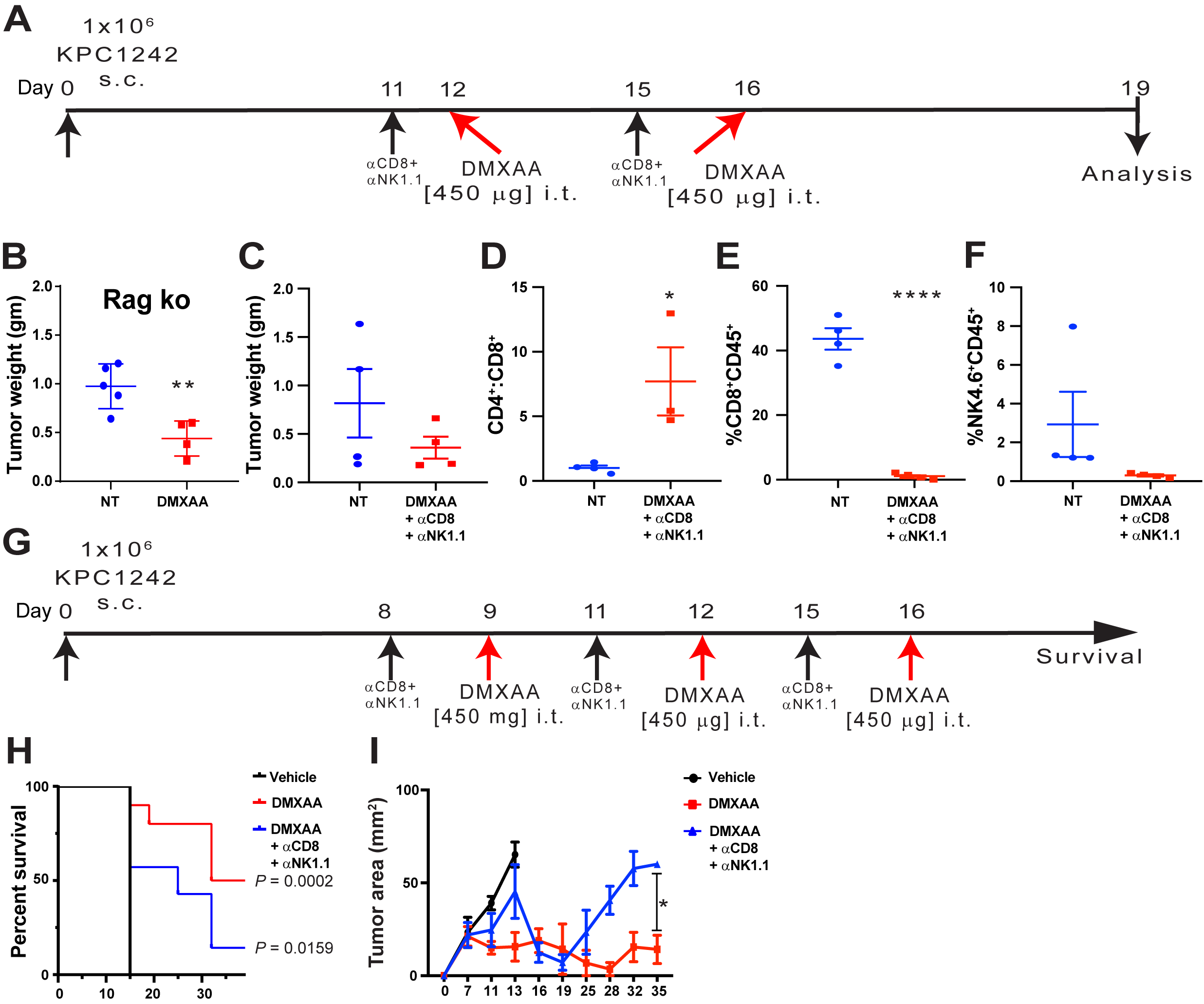

Supplement: Supplementary file 2 — Anti-tumor effects of STING agonist on αCD8/αNK1.1 pancreatic tumors. (A, G) Experimental treatment strategy of subcutaneous pancreatic tumors in C57BL/6 mice. Control (NT) or experimental mice were treated as indicated (B) Pancreatic tumor wet weights were measured from Rag-1 deficient mice that had been implanted with KPC1242 cells and left not treated as a control or treated with DMXAA (C). Tumors were collected 19 days after implantation and tumor weight measured. (D-F) Tumors were processed into single cell suspensions and CD4:CD8 ratios and percent CD8+ and CD4+cells within the CD45+ compartment determined by flow cytometry. n = 4 mice per group (H). Kaplan-Meier survival curves are shown for the indicated control and experimental groups. n = 7 mice per group. (I) Tumor growth over time in mice DMXAA-treated (red lines), DMXAA + αCD8/NK1.1 treated (blue lines) or NT control (black lines). *, P ≤ 0.05; **, P ≤ 0.01; ***, P ≤ 0.001; ****, P ≤ 0.0001. (TIF 582 kb) [file 40425_2019_573_MOESM2_ESM.tif]

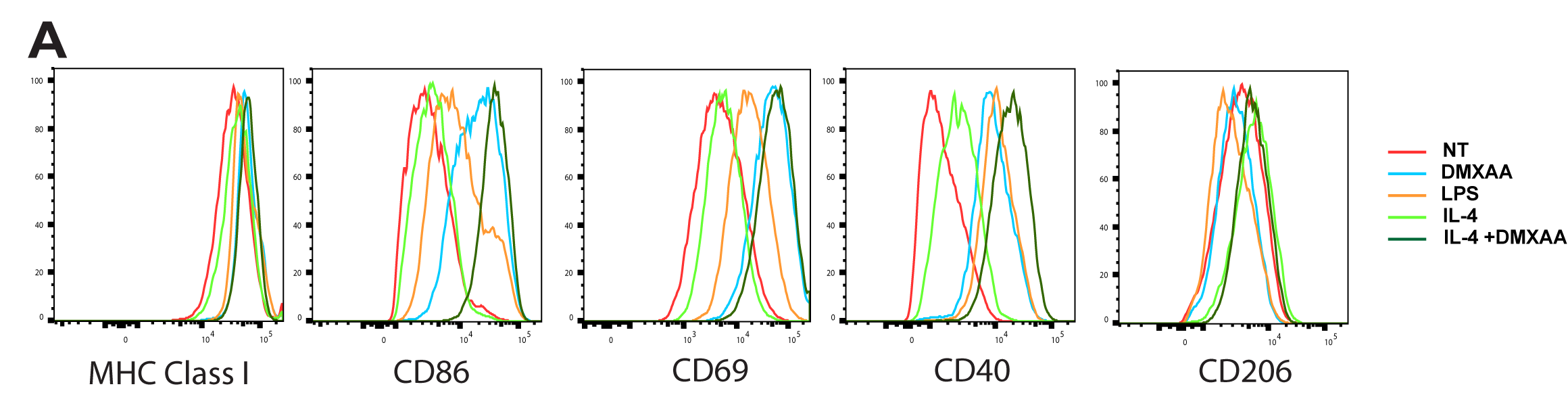

Supplement: Supplementary file 3 — Bone marrow-derived macrophage characterization. Isolated bone marrow–derived macrophages were cultured in G-CSF alone (M0) or polarize activated on day 7 by change fresh medium containing either 100 ng/ml LPS, to simulate M1 activation, or 10 ng/ml IL4 to induce M2 polarization for 48 h. Some M2 activated BMDMs were stimulated with 20 μg/mL DMXAA for 18 h and stained for flow cytometry analyses. (TIF 262 kb) [file 40425_2019_573_MOESM3_ESM.tif]
